# The development of My Care Hub Mobile-Phone App to Support Self-Management in Australians with Type 1 or Type 2 Diabetes

**DOI:** 10.1038/s41598-019-56411-0

**Published:** 2020-01-08

**Authors:** Mary D. Adu, Usman H. Malabu, Aduli E. O. Malau-Aduli, Bunmi S. Malau-Aduli

**Affiliations:** 1 College of Medicine and Dentistry, James Cook University, Townsville, Australia; 2 College of Public Health, Medical and Veterinary Sciences, James Cook University, Townsville, Australia

**Keywords:** Diabetes, Epidemiology

## Abstract

Non-adherence to self-management poses a serious risk to diabetes complications. Digital behavioural change interventions have the potential to provide education and motivate users to regularly engage with self-management of diabetes. This paper describes the development of My Care Hub mobile phone application (app) aimed at supporting self-management in people with type 1 or type 2 diabetes. The development of My Care Hub involved a comprehensive process of healthy behavioural change identification, end users’ needs, expert consensus, data security and privacy considerations. The app translation was a highly iterative process accompanied by usability testing and design modification. The app development process included: (1) behaviour change strategy selection; (2) users’ involvement; (3) expert advisory involvement; (4) data security and privacy considerations; (5) design creation and output translation into a smartphone app and (6) two usability testings of the app prototype version. The app features include self-management activities documentation, analytics, personalized and generalized messages for diabetes self-management as well as carbohydrate components of common foods in Australia. Twelve respondents provided feedback on the usability of the app. Initially, a simplification of the documentation features of the app was identified as a need to improve usability. Overall, results indicated good user satisfaction rate.

## Introduction

More than 1.1 million Australians have type 1 (12%) and type 2 (85%) diabetes^1^. Poorly controlled diabetes increases the risk of chronic complications^2^. Diabetes self-management education (DSME) and support are effective interventions to assist patients navigate through decision making processes for participation in self-management activities necessary for improved glycemic control^3^ and reduce the risk of developing complications^4^. Self-management support could be educational, behavioural, psychosocial or clinical^5^. Providing ongoing support to patients could mitigate disease related distress, improve adherence to recommended self-management activities and consequently enhance health outcomes^5,6^, but this practice has proven difficult in a real-world context^7^. Provision of self-management support through the use of smartphone may help address health system level limiters which impact negatively on the frequency and quality of self-management support patient receives. Such limitations include time constraints, limited access to care providers^8^ and partial cost reimbursement by third party health insurance^9^.

Smart phone is a ubiquitous technological device with more than 2 billion users worldwide^10^, and over 16% of 6 billion mobile subscriptions are smartphone subscriptions^11^. In Australia, the growth in smartphone usage has been sporadic, with approximately 84% smartphone users among the entire population of mobile phone users in 2018, which is an increase from 74% in 2014^12^. Most smart phone functionalities are aided by apps (software that are designed to run on smartphones), which could complement highly developed health care technologies and serve as supporting tools in many chronic disease management^13^. Various apps have been developed to enhance self-management of diabetes^14–18^. However, most apps have no reference to health behavioral change models or scientific evidence-based theories^14,15,17^. Additionally, considerations of target users’ preferences^14,15^, and data privacy input^16,17^ are minimal. Furthermore, some lack educational information which is a crucial component of diabetes care, to foster coping skills for ongoing self-management in patients and improved health (glycemic) outcomes^17,18^.

## Objective

Emerging evidence support the use of smartphone apps for diabetes self-management^14,15,18^. However, there remains paucity of information in relation to the description of the development processes and design of smartphone apps interventions, leaving unanswered questions about how to productively leverage apps for diabetes self-management^19^. Adequate description of the development of interventions could decrease waste in health research and enhance better organized synthesis of study results^19,20^. Vivid explanation of study process will promote wider dissemination of research findings and improve the rigour and quality control of published research^21^. In addition, explicit reporting of intervention development can allow external scrutiny of its plausibility when later used in trials and help evaluators and policy makers decide which context to prioritize for future replication^22^.

On the other hand, assessing usability occupies a central part of app development^23^. A well designed app with high usability positively influences users’ engagement (re-use rates)^24^ Conversely, poor usability is associated with low effectiveness and engagement^24,25^. To the best of the authors’ knowledge, there is no published full report of the systematic development of a diabetes smartphone app targeting Australian population before use in a full trial. Therefore, this paper reports a full description of the development and usability testing process of an app named “My Care Hub” prior to use in a pilot trial. My Care Hub was developed as an app to best meet the needs of type 1 and type 2 diabetes patients in the provision of preferred features and diabetes educational contents which could foster incremental knowledge gain, self-efficacy and motivate patients to actively engage with self-management activities.

## Methods

An initial systematic review^19^ by the authors highlighted the importance of six essential factors that needed consideration in the diabetes app development before actual use in a Randomized Controlled Trial (RCT). Specifically, the factors included involvement of users and clinical experts, health behavioural change theory employed, data security and privacy considerations, and pilot testing^19^. In practice, these steps overlap and are iterative. Herein, we describe how these steps were considered in the development of My Care Hub. The process of pilot testing is excluded because it is beyond the scope of the present paper. Additionally, this paper describes the features and functionalities available in My Care Hub and the usability testing of its prototype. All activities conducted during the pre-development, development and testing stage of the app are described.

## Ethical Consideration

This study was approved by the James cook University Human Research Ethics Committee (H7087). Informed consent was obtained from all participants involved in the usability testing of My Care Hub. All methods were performed in accordance with the relevant guidelines and regulations.

### Stage 1: Pre-development

#### Users’ involvement

A user centered design process was utilised in the development of My Care Hub. An iterative process addressing people’s needs and the enabling infrastructure to meet those needs constitute the central focus of a user centered design approach^26^. People with type 1 or type 2 diabetes were the primary target users of My Care Hub, hence their involvement in the development process, in order to gather information and ideas for appropriate design and educational content of the app. We performed a need analysis study among a multinational audience of people with type 1 or type 2 diabetes using a mixed methods study design^27^. The study elucidated diabetes patients’ app feature preferences and explored their recommendations for inclusion to promote engagement with My Care Hub diabetes app. The result indicated that both patient groups desired apps that featured documentation (blood glucose and physical exercise), advisory information (educational), analytics (view trends in behavioural and health data indicators), reminders (with actionable notifications) and food nutritional database. Furthermore, patients desired educational information on approaches to problem solving when blood glucose data were out of the clinically recommended range. Basic guideline information for diabetes self-management was perceived to be highly beneficial. Full details of the findings of the needs analysis study have been published^27^.

### Health behavioural change theory

Maximizing the potential efficacy of health behavioral change interventions in humans requires an understanding of the theoretical models or mechanisms that underpin such behavioral changes^28^. An extensive literature search of behavioural change theories^29^ was adopted to make informed decision in the development of My Care Hub. This provided the opportunity to identify the best interventional techniques (and the underlying theories) that are likely to be effective in a diabetes self-management app. The first technique considered was self-monitoring; an important intervention that fosters adherence to self-management and driven by self-efficacy construct of the social cognitive theory^30,31^. Self-efficacy refers to confidence in a person’s ability to take action required to implement situation specific behaviours in order to attain specific goals (health outcomes)^32^. Self-efficacy forms the “major basis for action”, occupies a pivotal regulatory role in the causal structure of self-management and perseverance to continue even when barriers are encountered^32^. Hence, improving self-efficacy through task related activities such as self-monitoring increases the confidence and persistence towards accomplishing the task^33^. On this basis, it *was* decided that My Care Hub should provide users with a platform to create daily entries and monitoring of their health behavioral activities.

Although self-monitoring is an important feature in behavioural interventions, patients’ adherence to self-monitoring alone is often low, especially when not accompanied by another intervention which could further enhance self-efficacy^34^. High attrition rates when self-monitoring activities are the only intervention features in diabetes apps have been reported^16,18,35^. Therefore, sole-reliance on self-monitoring features in an app may limit its effectiveness and use to only those who are personally motivated to self-monitor regularly. Consequently, it was considered important to include other essential interventional features such as provision of information and feedback in My Care Hub.

Components of educational information and feedback in health behavioural interventions are best developed using Information-Motivation-Behavioural Skills (IMBS) model^36,37^. The IMBS model has several constructs pertaining to patient adherence to recommended health behaviours. Primarily, the self-management information needs to be easily actionable in order to enhance optimal care. In addition, the information should provide basic knowledge about the relevant medical condition and effective management strategies. Users also need to be provided with encouraging and motivational feedback and recommendations in order to boost their confidence and enhance their ability to achieve desired health behavioural changes^38,39^. Reinforcing healthy behaviour significantly improves self-efficacy for participation in self-management activities^40^ Furthermore, inclusion of behavioural skills which ensure patients are provided specific strategies on how to perform the adherence behaviours (diabetes self-management activities- e.g. choosing appropriate foods, problem solving for low/high blood glucose) are important to enhance effective self-management. Lastly, IMBS model indicates that strategies aimed at increasing patients’ knowledge are prerequisites for behavioural change but not sufficient enough to sustain the change^41^. Therefore, such information should be coupled with motivation to increase the possibility of adherence.

The IMBS constructs were employed in the development of three educational information modules embedded in My Care Hub. These modules were: general information on diabetes management, automated feedback in response to logged blood glucose and carbohydrate contents of foods. These modules were aimed at improving patients’ knowledge, self-efficacy and provide specific directions for fostering patients’ adherence to ongoing self-management.

### Data security and privacy considerations

Several security features and privacy policies in the design and development of mobile health apps^42^ were followed. Authorization and authentication of users to assess the app was controlled, whereby the first user interface after the app download is a login screen. The screen requires a user to enter an assigned unique username (email address) and password in order to gain access to the app features. Authentication verifies user session handling for future research and ensures that the server is not vulnerable to injection attacks^43,44^. Protection of data transfer from My Care Hub to cloud storage was ensured through encryption which makes the data illegible, unusable and indecipherable to unauthorized persons^44^. My Care Hub was built on a proprietary personal health (PHR) platform using Firebase^45^ and it has no connection or interaction with third party health information systems. In accordance with the “Australian Privacy Principle”^46^, the services of an independent legal firm was employed to develop a comprehensive legal document pertaining to legal agreement, terms of use and privacy policy. The document describes how My Care Hub manages personal information, data collection, usage and storage. The legal document was incorporated into My Care Hub app for easy accessibility by users.

### Expert advisory group

An expert advisory group comprising diabetes educators, endocrinologists, health researchers and app developers worked as a team to make decisions regarding My Care Hub. Implications of the users’ needs analysis study findings and effectiveness of identified health behavioural change interventions were discussed by the team^27^. Results were prioritized, adjusted and refined before a final agreement on the selection of My Care Hub features, design and content in order to meet the requested needs of users as much as possible.

### Stage 2: Design and translation of pre-development output into a smartphone app

#### Development of My Care Hub Prototype

App developers within the eResearch Team at James Cook University (JCU), Queensland, Australia developed the prototype. The purpose of the app was to serve as a tool for monitoring self-management activities, providing access to information and aiding motivation to engage with diabetes self-management. These purpose were adequately met by providing opportunity to users to monitor and track their core health behaviours (weight, physical activities and carbohydrate contents of the foods consumed) and vitals (blood glucose); and have easy access to three streams of diabetes educational information: (i) an overview of diabetes management, (ii) feedback on logged blood glucose data, and (iii) information on carbohydrate contents in foods. To ensure that the app is usable by both types 1 and 2 diabetes patients, the feedback took into consideration the different recommended blood glucose values.

Initially, a visual skeletal framework of the app was created and subsequently used to develop the prototype. The framework comprised features, educational content, lay out, and interfacing of the app functionalities. The app was developed in Java in order to run on Android Platform. The resulting prototype was thoroughly examined and tested to ensure that all specified requirements were incorporated into the app for optimal functionality.

#### Features and Functionalities of My Care Hub

**Figure 1:**
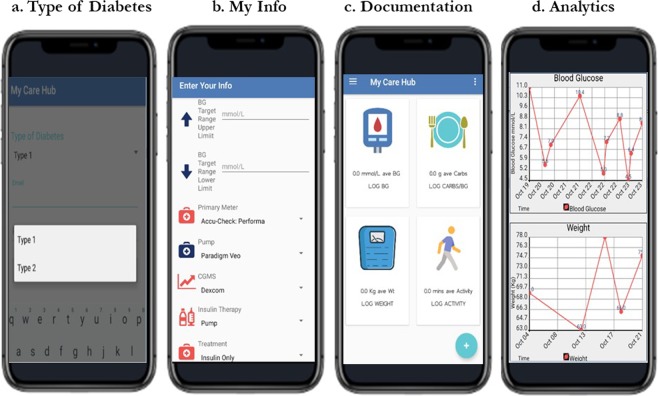
My Care Hub screenshots.

**Figure 2:**
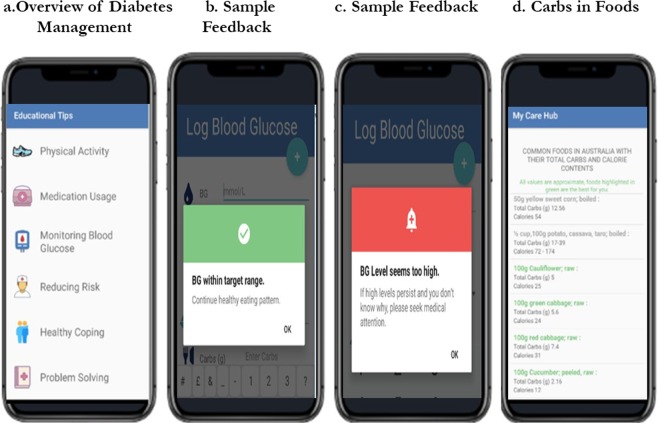
My Care Hub educational modules screen examples.

My Care Hub contains the following features: selection of type of diabetes, My Info, documentation, View insights, Carbs in foods and educational tips. Description of each of these features are provided below and a screen shot of the features in My Care Hub are provided in Figs. 1–2.

Type of DM: This feature is displayed after the user has downloaded, installed and registered the app on their smart phone. The user is then required to choose the type of diabetes they have (see Fig. 1a) in order to determine the type of feedback to be received when blood glucose data are logged.

My Info: This feature gives the user the choice to set up a profile to document their treatment details. Users can indicate their treatment specifics such as recommended blood glucose targets (upper and lower), types of medication (oral only, oral plus insulin, insulin only or none) and types of blood glucose meter, insulin pump, continuous glucose meter, if applicable. Profile set up may also include personal details such as gender and height (Fig. 1b).

Documentation: The home screen of My Care Hub (Fig. 1c), displays all its documentation features for logging of self-management activities including blood glucose, weight, physical activities and carbohydrate content of foods consumed. The actual date and time of logging is automatically set and users have the option to record the activity location. For the blood glucose (BG) interface, the type of BG data measured (either fasting or post hours post breakfast, lunch or dinner) can be selected from a drop menu and saved.

View Insights: This analytic feature provides users with graphical display of all data logged through the documentation features (Fig. 1d). It also takes the average of all data logged into each of the documentation features over a period of seven days and displays the mean on the homepage in form of summary feedback. This enables patients to visualise trends in lifestyle activities (especially physical activity and carbohydrate content of foods) and observe the impact on blood glucose levels over time^47^, thus fostering the ability to adjust their self-management strategy accordingly.

Educational modules: My Care Hub comprises three educational modules:

(a) Overview of diabetes management: The app contains textual information on the seven essential self-management activities for people with diabetes. These activities predict the following good health outcomes: lifestyle modifications (healthy eating and improved physical activity), monitoring of blood sugar, complying with medications, good problem-solving skills, healthy coping skills, and risk-reduction behaviours (such as smoking cessation and reduction in alcohol intake)^48^. (Fig. 2a). The messages were designed to be action-oriented, create awareness about associated benefits and provide engagement suggestions. The messages were written in plain language to enable users find, interpret and act on findings^49^. Table 1 depicts examples of app messages under each of the vital self-management activities.

**Table 1:** Examples of app messages.

Category	Message
What is diabetes?	Diabetes results from inherited and/or acquired deficiency in insulin production or by the ineffectiveness of the insulin produced.
	In a diabetic state, insulin function is impaired, therefore the body needs conscious help to manage blood sugar by eating right, exercising, taking medications appropriately and reducing stress.
Health food choices	Stay motivated to eat healthy. When new foods are eaten, testing of blood sugar before and 2 hours after the first bite is recommended in order to see the effect of the food on the blood sugar.
	Vegetables are rich in fibre, make you comfortably full, have vitamins for healthy immune system and most likely not spike your blood sugar level.
Physical activity	Exercise improves bone strength, keeps the heart and blood vessels healthy and lowers insulin resistance.
	Exercise does not have to involve the whole day. You can split it up into 10 minutes, 3 times a day at a convenient period for you.
	If you are on insulin and planning for prolonged exercise, if your blood sugar level is below 6 mmol/L, it is advisable to eat an extra carbohydrate.
Medication usage	Some diabetes medicines can lose effectiveness if they are old or stored improperly. For example, insulin should not be frozen or exposed to extreme heat.
	If you often forget to take your medications, setting a reminder or alert may help you.
	Do not inject insulin on moles or scars because it slows absorption rate and limit insulin effectiveness.
Monitoring blood glucose	Some of the important times to test blood glucose include before breakfast (fasting), 2 h after a meal, before rigorous exercise, before bed, when you are not feeling well.
	Regular testing of blood sugar improves confidence to look after diabetes, give a better understanding of the impact of food intake, medication, exercise and other factors such as stress and illness on blood glucose.
Reducing the risk of complications	Keeping appointment with the eye/foot doctor, endocrinologist and other health care team is a great way to detect on time any complication development.
	Smoking increases the risk of developing acute and chronic complications in diabetes mellitus.
	Keep your foot healthy by checking regularly for any changes, washing daily and wear shoes that fit properly. In the occurrence of unexplainable blisters, cuts or openings, it is best to consult your doctor.

(b) Feedback: My Care Hub includes an algorithm that provides automated feedback messages in response to logged blood glucose (BG) data. It assesses if the logged BG data are within or outside the clinically recommended range and provides appropriate encouragement or advice. This feature was founded on relevant evidence-based literature and Diabetes Australia recommended BG level guidelines^50^ for appropriate and accurate feedback. For people with type 1 diabetes; 4–8 mmol/L at fasting and <10 mmol/L 2 hours post prandial BG levels were recommended. Fasting levels of 6–8 mmol/L and 2 hours post prandial levels of 6–10 mmol/L were endorsed for people with type 2 diabetes.

A range of evidence-based, motivational, health promotional and behavioural skills information were developed as feedback on BG levels recommended for both type 1 and type 2 diabetes. Decision based system rules were programmed into the app to ensure that users receive semi-individualized feedback based on their logged data. The system is controlled by the indicated type of diabetes, value of BG (whether within or beyond the clinically recommended range) and the period of BG measurement (either fasting or 2 hours post prandial). Once BG data are logged, the app delivers a brief feedback. If the BG is within the clinically recommended range, the app delivers messages to encourage the patient to continue with regular self-management. Such messages include: “Excellent; BG within target range, continue your medication as prescribed”, and “Excellent, BG within target range, continue with healthy eating”). It has been demonstrated that providing motivation and encouragement after attaining a goal can enhance self-efficacy^40^ which in turn, facilitates health behavioural changes in disease control^51^. If the logged BG are outside normal range, the app offers suggestions related to problem solving for low or high BG as deemed appropriate. For instance, if logged BG is less than 4 mmol/L, sample feedback includes: “BG levels seems too low, this may occur when medication is not balanced with food and physical activities”, and “you are at risk of hypoglycemia; treat immediately”. If BG values are extremely aberrant to the normal range (over 15 mmol/L), messages such as “if high levels persist and you don’t know why, seek medical attention immediately” are triggered. Messages are unidirectional and displayed using colour labels, where green, orange and red indicate “ideal”, “not ideal” and “extremely low or high” BG levels, respectively (Fig. 2b,c)

(c) Carbohydrates in foods: This feature comprises textual information about carbohydrate and calorie components of common foods in Australia sourced from the Australian Food, Supplement and Nutrient database (AUSNUT 2011–13)^52^. Some commonly available foods were selected for this database and organised under four main groups: fruits and vegetables; eggs and meat; diary; and legumes and grains. Portion sizes and approximate carbohydrate and calorie content were provided for each food item. For example, 1 slice (40 g) of whole wheat bread contains 20.56 g of carbohydrates and 111 calories. Foods with low glycemic index were displayed in green colours as healthier models for consumption by those who have diabetes^53^ (Fig. 2d).

Functionalities and Features for future research: To facilitate future research, export of the logged data into a cloud storage for all documentation features, date and time of log-ins were enabled for downloading as comma-separated value files into our database for subsequent statistical analysis. Furthermore, the app platform offers analytics of users’ logged data and performance optimization and includes cloud and in-app messaging features to allow for push notifications. These features will provide additional educational messages for future research.

### Stage 3: Usability testing

#### Usability testing of My Care Hub was done in two stages

(1) Members of the public who do not have diabetes were randomly selected for early testing strategy to ascertain the technical performance of the app and to identify any navigation issues when downloaded on various android phones. The app’s functionality and aesthetic usability testing by users was over a period of 7 days. Using convenient and snow balling sampling methods, twelve participants (app testers) were individually contacted and provided with information about My Care Hub and the ultimate aim of its development. The primary inclusion criterion was access to an android phone since the app was developed on an android platform only. Specific tasks required of the app testers included app download and registration; daily log-ins of random numbers into the documentation features (no limit was set to the amount of data to be logged into the app); a read through the educational information about diabetes self-management embedded in the app; observation of the automated feedback messages in response to each logged BG; examination of the graphical outputs of all the documentation features; and general browsing of the app. Any data crash or lag time in the app response during launching and usage, screen “swiping”, using the slide out key board and apps touch screen buttons were also monitored. Each tester was contacted via email and provided with a unique user name, password and app download instruction. Only testers who signed into and used the app were provided with a link to the online survey questionnaire items adapted from the mobile app rating scale^54^. Testers were asked to rate the app’s functionality (performance, ease of use, navigation, gestural design) and aesthetics (layout, graphics, visual appeal of the analytic display). The results from this first stage of usability testing was used to improve the app prototype before the second stage was completed.

(2) The second stage of testing recruited participants who had diabetes from the diabetes center of a tertiary hospital in Queensland, Australia, and through snow balling. Participants were asked to use My Care Hub and provide feedback. In addition to functionality and aesthetics of the app, they also provided feedback on their satisfaction using measures that included perceived usefulness of the app to motivate participation in, and increase awareness of, diabetes self-management, intention to use, perceived ease of use and accuracy of the educational components of the app. Furthermore, they were asked if they would recommend the app to people with type 1 or type 2 diabetes and give an overall rating of 1–5 with “1 = one of the worst apps I have ever used” and “5 = one of the best apps I have ever used”. The usability questions are provided in the Supplementary Information.

## Results

### Demographic and health characteristics

Of the 12 testers without diabetes, only eight (8) signed into the app. In the second stage of testing, 6 individuals with type 1 or type 2 agreed to test the app, but only 4 signed in. Time constraint was cited by the 6 people who withdrew from the testing as reason for non-participation. Feedback was provided by the remaining 12 participants (8 non diabetes + 4 with diabetes) who downloaded and used the app. The mean age of participants was 43.08 ± 14.02 years (range 28–76 years) and 58% of them were women. More than 75% were married and had obtained an educational level of first degree or higher. Of the four participants who have diabetes, three of them had type 2, one of them was diagnosed less than 5 years ago, while the remaining 3 had been diagnosed over 5 years. None of them reported hypoglycemia unawareness. Three of the four participants reported that their recommended range of fasting blood glucose was 4 to 7 mmol/L, with a post prandial of 5 to 9 mmol/L. The remaining one participant who had type 2 diabetes did not provide any information on this.

### Usability result

In stage one of testing, all participants but one, were able to easily learn the use of the app following instructions provided via email. This one participant expressed an initial difficulty in operating the app by providing free text comment in the survey. Locating and learning how to use the documentation features took time, hence the suggestion to include a video recording of the instructions in addition to the already developed instruction manual for future users. Although, it was only one participant that indicated difficulty in using the app, it meant 13% of the participants had potential usability issues in relation to layout and ease of use. Therefore, based on the results of the first stage usability testing, minor modifications were made. In the prototype, accessing the documentation features required users to tap on a menu bar located on the top left corner of the app’s homepage (which may not be apparent to users). The current version provides an improvement with an additional access via the app’s homepage through direct taping on the icons of the documentation features.

In the second stage of testing using individuals with type 1 or type 2 diabetes, all the four participants were able to use the app easily. Across all participants (12), majority were satisfied with the performance of the app in terms of how fast the app features and components work (50% chose “perfect/timely response”, 41.7% “mostly functional”, 8.3% “app work overall, slow at times”). Several participants were able to navigate between app screens and features easily (66.7% “perfectly logical and clear screen flow throughout”, 25% “easy to use”) and felt that interactions across all tabs in the app were consistent and spontaneous (41.7% “perfectly consistent and spontaneous”, 50% “mostly consistent and spontaneous”). Many participants were satisfied with the arrangement and size of icons/contents in the app (66.7% “mostly clear”, 33.4% “professional, simple, clear and logically organised”) and the quality of graphics was high/very high. With regards to the general visual appeal of the app and the analytic feature, 83.3% participants chose very high/high level of visual appeal for both domains. The app was given a five and four star rating by 8.3% and 91.7% respondents respectively.

Among the four participants with diabetes, 75% of them strongly agreed that they saw value in the educational content of the app as it was relevant and likely to raise awareness of the importance of diabetes self-management. 75% strongly agreed that the app is likely to increase motivation of people with diabetes to engage in self-management activities. Furthermore, 75% noted that they would recommend My Care Hub to people with type 1 or type 2 diabetes and that they can continue to use it if granted continual access to the app.

## Discussion

This paper follows the principle of intervention development study report which details “the rationale, decision making processes, methods and findings from the inception of the intervention to the usability testing prior to full trial or evaluation”^55^. To the best of the authors’ knowledge, My Care Hub is arguably the first diabetes self-management app aimed at Australian population with type 1 or type 2 diabetes to have reported/ documented a systematic and transparent approach to its development based on empirical and theoretical framework of health behavior change theories, involvement of users and clinical experts, data security and privacy considerations^19^.

Behavioural theory is critical to the development of health behavioural change interventions^56^ because interventions grounded in theory are more effective at modifying behaviour^29,57^. Health behavioural theories predict how applying an intervention will drive change in underlying behavioural mechanisms or technique (mediating construct) that will in turn, drive behavioural change (output)^29^. Majority of currently available diabetes app are lacking in health behavioural content^19,58^ which may be an early indication of the low potential of such apps to influence behaviour long term^57^. My Care Hub is grounded in two major theories of behavioural change: Social cognitive theory^31^ and Information-Motivation-Behavioural Skills model^36,37^. Diverse constructs were employed within these theories; hence, giving My Care Hub the potential for effectiveness when eventually used as an intervention in RCT.

Specific features and educational content in My Care Hub were chosen based on users’ needs and preferences^27^. This is an added strength of the app which is often lacking in many health apps that are developed without considering the needs of the end users or guidelines for the management of such diseases^59,60^. Development of an intervention to meet the actual needs and demands of targeted users assures the feasibility of the product^26^. Previous reports show that only few diabetes mobile apps incorporate elements of clinical best practices established by diabetes professionals^61^. Blood glucose level guidelines recommended by Diabetes Australia were consulted during the development of feedback messages provided in My Care Hub in order to ensure clinically sound information is provided to the patients. Providing relevant feedback is an important strategy to stimulate reflection in patients about their blood glucose goal and to engage them in healthy behaviours necessary for optimal health outcomes^62^.

Data privacy and security in mobile health interventions are vital to intervention development, relevance and acceptance of such technology^63^, therefore, health apps must be protected from security breaches. This was ensured in My Care Hub. The multi-stage usability evaluation method applied to My Care Hub have better propensity to capture the complete usability of a technology in comparison to a single method^64,65^. First stage of testing revealed important changes to the app design in order to improve the ease of access to user interface. Similar to our findings, participants in studies assessing the usability of mobile apps for diabetes self-management had commented positively on the performance and ease of navigation of the app^65,66^. Furthermore, good level of satisfaction reported by our participants in relation to the app’s graphics, layout and visual appeal has also been reported in previous studies^65,67^. End users saw value in the app as a tool to support diabetes self-management and expressed interest in continuing to use the app in the future.

### Strengths and limitations

The strengths of this study include a full description of the development process of My Care Hub - an app aimed at the Australian population with type 1 or type 2 diabetes as opposed to the scanty reports of only efficacy and effectiveness of diabetes apps within RCTs in the published literature^19^. In our systematic review of previous RCTs using a diabetes app as an intervention, none of the studies provided detailed information about their developmental process^19^. The need for full description of the intervention development strategy, including detailed explanation of how information gathered from users was used to guide the development process has been met in our current study. Not only is the use of two distinct approaches to usability testing unique in providing effective development guide and strategies for future apps, the results from both phases also allow for identification of pertinent issues from a general and targeted populace of app users. Usability testing of the app at this stage and not later during feasibility testing, is essential for identifying improvement needs prior to full studies to secure future engagement with My Care Hub by patients with diabetes.

A limitation of this study is that the participants in the usability study may not be representative of the target population due to low sample size. Nonetheless, studies have shown that large sample sizes are not required in usability studies, as they do not propose inferential results. Therefore, the number of participants involved in the usability testing of My Care Hub in this study, may have been sufficient to identify usability issues in the app which may occur under conditions of regular use^68^. Another limitation is that only some of those who initially agreed to test the app eventually did so. This may have introduced a selection bias wherein only participants interested in the use of an app downloaded and signed into it and provided feedback. It is possible that we could have drawn different conclusion if all the initial participants had used the app. However, in reality, research outcomes are not free of either opinion or bias because they are highly subjective^69^. In addition, although, the app needs analysis results indicated that patients will like an app that reminds them of their self-management activities, currently My Care Hub app does not have this feature. This is because upon prioritization and consideration, we felt inclusion of a reminder feature might further increase the complexity of the app. In its’ present version, the app entails a variety of features, therefore an addition of reminder feature (which ideally will be needed for each of the diabetes specific tasks) will definitely increase the app’s complexity. Studies have reported a significantly negative correlation between number of app features and ease of use^70^. Nonetheless, upon ensuring that the app remains user friendly, inclusion of a reminder feature is a possible consideration for our future research. Lastly, given that no data was collected about the specifics of participants’ android smartphone configurations, we were unable to assess the influence of such configuration on their responses to My Care Hub usability testing.

### Future research

Future research includes pilot testing of My Care Hub as a key step for optimizing an intervention before its evaluation in a full randomized controlled trial^71^. This pilot testing will provide valuable information on users’ engagement, acceptability and preliminary efficacy^72^ as well as the development of future versions of the app (if required) and design of future trials^73^.

My Care Hub is currently only available for Australians on Android operating system. The decision to make the app Australia-specific was based on the differences in the guidelines for recommended blood glucose levels between countries. There was the need to focus resources on developing a native app that could as much as possible provide a better user experience instead of including additional country specific requirements. My Care Hub presents a proof of concept and can be developed for other nations in order to reach a wider proportion of users, but there will be the need to modify the Australia-specific features of the app before it could be used elsewhere.

## Conclusions

Detailed explanation of My Care Hub development was provided for future researchers to learn and understand the development process. My Care Hub has the potential to benefit Australians with types 1 and 2 diabetes in monitoring self-management activities. The app provides easy access to educational information which could enhance knowledge and motivate patients to perform self-management activities to improve glycemic control. An app such as My Care Hub developed based on the needs and preferences of its intended users maximizes the potential to enhance self-management.

## Supplementary information

Supplementary Information

## References

[1] Australian Institute of Health and Welfare. Cardiovascular disease, diabetes and chronic kidney disease: Australian facts: prevalence and incidence: Cardiovascular, diabetes and chronic kidney disease. CKD 2 (2). https://www.aihw.gov.au/reports/heart-stroke-vascular-disease/cardiovascular-diabetes-chronic-kidney-prevalence/contents/table-of-contents (2019).

[2] Alberti KGMM, Zimmet PF (1998). Definition, diagnosis and classification of diabetes mellitus and its complications. Part 1: diagnosis and classification of diabetes mellitus. Provisional report of a WHO consultation. Diabe Med..

[3] Heinrich E, Schaper NC, de Vries NK (2010). Self-management interventions for type 2. diabetes: a systematic review. Eur Diab Nurs..

[4] Powers MA (2015). Diabetes Self-management Education and Support in Type 2 Diabetes: A Joint Position Statement of the American Diabetes Association, the American Association of Diabetes Educators, and the Academy of Nutrition and Dietetics. Diabetes Care..

[5] Haas L (2012). National standards for diabetes self-management education and support. Diab Educ..

[6] Norris SL, Lau J, Smith SJ, Schimid. CH, Engelgau MM (2002). Self-management education for adults with type 2 diabetes: a meta-analysis of the effect on glycemic control. Diabetes Care..

[7] Kennedy, A. *et al*. Implementation of a self-management support approach (WISE) across a health system: a process evaluation explaining what did and did not work for organisations, clinicians and patients. *Implement Sci*. **9**(1), 129; 10.1186s/13012-014-0129-5 (2014). 10.1186/s13012-014-0129-5 PMC4210530 25331942

[8] Wilkinson A, Whitehead L, Ritchie L (2014). Factors influencing the ability to self-manage diabetes for adults living with type 1 or 2 diabetes. Int J Nurs Stud..

[9] Piette JD (2007). Interactive behavior change technology to support diabetes self-management: where do we stand?. Diabetes Care..

[10] Statistica. Number of smartphonr users wordwide from 2014 to 2020 (in billions). https://www.statista.com/statistics/330695/number-of-smartphone-users-worldwide/ (2019).

[11] MobiThinking. Global mobile statistics 2014 part A: Mobile subcribers; handset market share; mobile operatiors. http://mobiforge.com/research-analysis/global-mobile-statistics-2014-part-a-mobile-subscribers-handset-market-share-mobile-operators (2019).

[12] Statista. Share of mobile phone ussers that use a smartphone in Australia from 2014 to 2019. https://www.statista.com/statistics/257041/smartphone-user-penetration-in-australia/ (2019).

[13] Kay, M., Santos, J. & Takane, M. mHealth: New horizons for health through mobile technologies. *WHO*. **64** (7), 66–71, https://apps.who.int/iris/handle/10665/44607 (2011).

[14] Waki K (2014). DialBetics: a novel smartphone-based self-management support system for type 2 diabetes patients. J Diabetes Sci Technol..

[15] Quinn CC (2011). Cluster-randomized trial of a mobile phone personalized behavioral intervention for blood glucose control. Diabetes Care..

[16] Kirwan Morwenna, Vandelanotte Corneel, Fenning Andrew, Duncan Mitch J (2013). Diabetes Self-Management Smartphone Application for Adults With Type 1 Diabetes: Randomized Controlled Trial. Journal of Medical Internet Research.

[17] Kim HS (2014). Efficacy of the smartphone-based glucose management application stratified by user satisfaction. Diabetes Metab J..

[18] Kim Hun-Sung, Choi Wona, Baek Eun Kyoung, Kim Yun A, Yang So Jung, Choi In Young, Yoon Kun-Ho, Cho Jae-Hyoung (2014). Efficacy of the Smartphone-Based Glucose Management Application Stratified by User Satisfaction. Diabetes & Metabolism Journal.

[19] Adu Mary D, Malabu Usman H, Callander Emily J, Malau-Aduli Aduli EO, Malau-Aduli Bunmi S (2018). Considerations for the Development of Mobile Phone Apps to Support Diabetes Self-Management: Systematic Review. JMIR mHealth and uHealth.

[20] Hoffmann TC (2017). Enhancing the usability of systematic reviews by improving the consideration and description of interventions. BMJ..

[21] Munafò MR (2016). Opening up addiction science. Addiction..

[22] Moore GF (2015). Process evaluation of complex interventions: Medical Research Council guidance. BMJ..

[23] Zaid B, Jamaludin R, Wafaa B (2012). A comparative study of usability methods for mobile applications. Int J Sci Eng Res..

[24] Eysenbach Gunther (2005). The Law of Attrition. Journal of Medical Internet Research.

[25] Whitlock Laura A., McLaughlin Anne Collins (2012). Identifying Usability Problems of Blood Glucose Tracking Apps for Older Adult Users. Proceedings of the Human Factors and Ergonomics Society Annual Meeting.

[26] International Organisation for Standardization. Egornomics of human system interaction-Part 210: Human center design for interactive systems. https://www.iso.org/standard/52075.html

[27] Adu Mary D., Malabu Usman H., Malau-Aduli Aduli E. O., Malau-Aduli Bunmi S. (2018). Users’ preferences and design recommendations to promote engagements with mobile apps for diabetes self-management: Multi-national perspectives. PLOS ONE.

[28] Davis R, Campbell R, Hildon S, Hobbs L, Michie S (2015). Theories of behaviour and behaviour change across the social and behavioural sciences: a scoping review. Health Psychol Rev..

[29] Michie S, Johnston M, Francis J, Hardeman W, Eccles M (2008). From theory to intervention: mapping theoretically derived behavioural determinants to behaviour change techniques. Appl Psychol..

[30] Bandura A (2001). Social cognitive theory: An agentic perspective. Annu Rev Psychol..

[31] Haffernan C (1988). *Social foundations of thought and action: A social cognitive theory*, Albert Bandura Englewood cliffs. Prentice Hall. Behav Change..

[32] Bandura, A. *Self-efficacy: The Exercise of Control*. (Freeman, (1997).

[33] Barling J, Beattie R (1983). Self-efficacy beliefs and sales performance. J Organ Behav Manage..

[34] Mishali, M., Omer, H. & Heymann, A. The importance of measuring self-efficacy in patients with diabetes. *Fam Pract*. **28**(1),82-87;19.1993/fampra/cmq086 (2010). 10.1093/fampra/cmq086 21047940

[35] Guertler D, Vandelanotte C, Kirwan M, Duncan MJ (2015). Engagement and nonusage attrition with a free physical activity promotion program: the case of 10,000 steps Australia. JMIR..

[36] Fisher WA, Kohut T, Schachner H, Stenger P (2011). Understanding self-monitoring of blood glucose among individuals with type 1 and type 2 diabetes. Diabetes Educ..

[37] Fisher, W. A., Fisher, J. D. & Harman, J. The information-motivation-behavioral skills model: A general social psychological approach to understanding and promoting health behavior In *Social psychological foundations of health and illness*. (ed. Suls, J. & Wallston, K. A.) **82**, 106 (Balckwell, (2003).

[38] De Bruijn GJ (2010). Understanding college students’ fruit consumption. Integrating habit strength in the theory of planned behaviour. Appetite..

[39] Bandura, A. Self-effficacy In *Encyclopedia of Human Behavior* (ed. Ramachaudran, V. S). **4**; 71–81. (Academic, (1994).

[40] Williams SL, French DP (2011). What are the most effective intervention techniques for changing physical activity self-efficacy and physical activity behaviour—and are they the same?. Health Educ Res..

[41] Mazzuca SA (1982). Does patient education in chronic disease have therapeutic value?. J Chronic Dis..

[42] Morera EP (2016). Security recommendations for mHealth apps: Elaboration of a developer’s guide. J Med Syst..

[43] Lin, J. C. & Chen, J. M. The automatic defense mechanism for malicious injection attack In *7th IEEE International Conference on Computer and Information Technology*. (2007).

[44] Osawaru ER, A.H RA (2014). A Highlight of Security Challenges in Big Data. Int J Inform Syst Eng..

[45] Moroney, L. *The Definitive guide to firebase: build Android apps on Google’s mobile platform*. (Apress (2017).

[46] Australian Government, office of the Australian Information Commissioner. Guide to developing an APP privacy policy. https://www.oaic.gov.au/agencies-and-organisations/guides/guide-to-developing-an-app-privacy-policy (2019)

[47] Winters-Miner, L.A. Seven ways predictive analytics can improve healthcare. https://www.elsevier.com/connect/seven-ways-predictive-analytics-can-improve-healthcare (2019).

[48] Tomky D (2008). Aade Position Statement; AADE7TM Self-Care Behaviors. Diabetes Educ..

[49] Stableford S, Mettger W (2007). Plain language: a strategic response to the health literacy challenge. J Public Health Policy..

[50] Diabetes Australia. Blood glucose monitoring. https://www.diabetesaustralia.com.au/blood-glucose-monitoring (2015).

[51] Rgn AH, Rgn HEW (2002). Role of self-efficacy and behaviour change. Inter J Nurs Pract..

[52] Australian NewZealand. Food Standards. Astralian Food, Supplement and Nutrient (AUSNUT) database 2011–13. http://www.foodstandards.gov.au/science/monitoringnutrients/ausnut/foodnutrient/Pages/default.aspx (2019).

[53] Rizkalla SW, Bellisle F, Slama G (2002). Health benefits of low glycaemic index foods, such as pulses, in diabetic patients and healthy individuals. Br J Nutr..

[54] Stoyanov SR (2015). Mobile app rating scale: a new tool for assessing the quality of health mobile apps. JMIR Mhealth and Uhealth..

[55] Hoddinott P (2015). A new era for intervention development studies. Pilot Fasibility Stud..

[56] Campbell NC (2007). Designing and evaluating complex interventions to improve health care. BMJ..

[57] Craig, P. *et al*. Developing and evaluating complex interventions: the new Medical Research Council guidance. *BMJ*. **337**; 10.1136/bmj.a1655 (2008). 10.1136/bmj.a1655 PMC2769032 18824488

[58] Cowan LT (2013). Apps of steel: are exercise apps providing consumers with realistic expectations? A content analysis of exercise apps for presence of behavior change theory. Health Educ Behav..

[59] Chomutare Taridzo, Fernandez-Luque Luis, Årsand Eirik, Hartvigsen Gunnar (2011). Features of Mobile Diabetes Applications: Review of the Literature and Analysis of Current Applications Compared Against Evidence-Based Guidelines. Journal of Medical Internet Research.

[60] Holtz B, Lauckner C (2012). Diabetes management via mobile phones: a systematic review. Telemed J E-Health..

[61] Brandell B, Ford C (2013). Diabetes professionals must seize the opportunity in mobile health. J Diabetes Sci Technol..

[62] Lie SS, Karisen B, Niemiec CP, Graue M, Oftedal B (2018). Written reflection in an eHealth intervention for adults with type 2 diabetes mellitus: a qualitative study. Patient Prefer Adherence..

[63] Wilkowska W, Ziefle M (2012). Privacy and data security in E-health: Requirements from the user’s perspective. Health Informatics J..

[64] Cho H (2018). A multi-level usability evaluation of mobile health applications: A case study. J Biomed Inform..

[65] Georgsson M, Staggers N (2016). An evaluation of patients’ experienced usability of a diabetes mHealth system using a multi-method approach. J Biomed Inform..

[66] Booth Alison O., Lowis Carole, Hunter Steven J., Dean Moira, Cardwell Chris R., McKinley Michelle C. (2016). Development and Evaluation of a Computer-Based, Self-Management Tool for People Recently Diagnosed with Type 2 Diabetes. Journal of Diabetes Research.

[67] Schmocker KS, Zwahlen FS, Denecke K (2018). Mobile App for Simplifying Life With Diabetes: Technical Description and Usability Study of GlucoMan. JMIR Diabetes..

[68] Macefield, R. How to specify the participant group size for usability studies: a practitioner’s guide. *Journal of Usability Studies*. **5**(1), 34–45, https://uxpajournal.org/how-to-specify-the-participant-group-size-for-usability-studies-a-practitioners-guide/ (2009).

[69] Six, J. M & Macefield R. How to determine the right number of participants for usability studies. https://webcache.googleusercontent.com/search?q=cache:T22AfzJmpC0J:https://www.uxmatters.com/mt/archives/2016/01/how-to-determine-the-right-number-of-participants-for-usability-studies.php+&cd=1&hl=en&ct=clnk&gl=au (2019).

[70] Arnhold M, Quade M, Kirch W (2014). Mobile applications for diabetics: a systematic review and expert-based usability evaluation considering the special requirements of diabetes patients age 50 years or older. JMIR..

[71] Eldridge SM (2016). Defining feasibility and pilot studies in preparation for randomised controlled trials: development of a conceptual framework. PLoS One..

[72] Feeley N (2009). The importance of piloting an RCT intervention. Can J Nurs Res..

[73] Day TL, Bench SD, Griffiths PD (2015). The role of pilot testing for a randomised control trial of a complex intervention in critical care. J Res Nurs..

