# Supplementary material for: The development of My Care Hub Mobile-Phone App to Support Self-Management in Australians with Type 1 or Type 2 Diabetes
Source: Sci Rep. 2020 Jan 8;10:7. doi: 10.1038/s41598-019-56411-0 (PMC6949290; doi:10.1038/s41598-019-56411-0)
Supplement: Supplementary file 1 — Supplementary Information [file 41598_2019_56411_MOESM1_ESM.pdf]

The development of *My Care Hub* Mobile-Phone App to Support Self-Management in Australians with Type 1 or Type 2 Diabetes.

Mary D. Adu, Usman H. Malabu, Aduli E.O. Malau-Aduli, Bunmi S. Malau-Aduli

Supplementary Information: My Care Hub Usability Questionnaire

Introductions

Welcome to this survey and thank you in advance for your interest to participate.

This survey aims to examine the usability (how easy and pleasant the features) of a newly developed mobile app (My Care Hub) to support diabetes self-management. The outcome of this survey will be of great value to determine the area of improvement which may be required in the app. The survey should take approximately 10 minutes. Your responses including your demographic information will remain anonymous. Participation is completely voluntary.

Instructions on completing the survey

Please read and consider the following questions carefully. Your candid responses are essential to ensure the reliability of this study.

Section A: Demographic

Below are questions about your background. Please pick one response for the multiple choice question

1. **What is your gender?**

   ☐ Male ☐ Female ☐ Others (Pls. specify) ……………..

2. **How old are you?** _____________(years)

3. **What is the highest level of education you have completed?**

   ☐ High school or equivalent ☐ Technical or vocational education

   ☐ College ☐ Bachelor degree ☐ Post graduate degree

   ☐ No formal education ☐ Others

4. **What type of diabetes do you have?**

   ☐ Type 1 ☐ Type 2 ☐ I don't have diabetes (please move to section B) ☐ Others (Please specify) ………………………….

5. **How long have you been diagnosed with diabetes (years)?**

   ☐ < 1 ☐ 1- 5 ☐ 6 - 10 ☐ 11 -15 ☐ ≥ 15 years ☐ Never

6. **What is the present fasting blood glucose range recommended by your health care provider? Please input your response in mmol/L. For example 4 to 6 mmol/L**

   ……………………………………………………..

7. **What is the present fasting blood glucose range recommended by your health care provider? Please input your response in mmol/L. For example 6 to 10mmol/L.**

   ……………………………………………………

Section B

The next set of questions are related to your experiences with the use of My Care Hub App over the last 7 days. Please answer each question based on your experience with the app. Your candid responses are essential to ensure the reliability of this information.

Functionality

1. **Performance**: How accurate/ fast do My Care Hub features (functions) and components (buttons/ menu) work?

1. App is broken; no/ insufficient/inaccurate response (e.g crashes/bugs/broken features e.t.c)
2. Some functions work, but lagging or contains major technical problems
3. App works overall. Some technical problems need fixing/Slow at time
4. Mostly functional with minor/negligible problems
5. Perfect/timely response; no technical bugs found

2. **Ease of use:** How easy is it to learn how to use My Care Hub; how clear are the menu labels/icons?

1. Menu labels/icons are confusing; complicated
2. Useable after a lot of time/effort
3. Usable after some time/effort
4. Easy to learn how to use the app
5. Able to use app immediately; intuitive; simple

3. **Navigation:** Is moving between My Care Hub screens logical/accurate/appropriate/uninterrupted; are all necessary screen link present?

1. Different sections within My Care Hub app seem logically disconnected and random/confusing/navigation is difficult
2. Usable after a lot of time/effort
3. Usable after some time/effort
4. Easy to use or missing a negligible link
5. Perfectly logical, easy, clear and intuitive screen flow throughout or offers shortcut

4. **Gestural design:** Are interaction (taps/scrolls) consistent and spontaneous across all components/ screens of My Care Hub?

1. Completely inconsistent/confusing
2. Often inconsistent/confusing
3. OK with some inconsistencies/confusing elements
4. Mostly inconsistent/spontaneous with negligible problems
5. Perfectly consistent and spontaneous

Aesthetics

5. **Layout:** Is arrangement and size of buttons/icons/menus/content on the apps' screen appropriate?

1. Very bad design, cluttered, some options impossible to select/locate/see/read device display not optimized
2. Bad design, random, unclear, some options difficult to select/locate/see/read
3. Satisfactory, few problems with selecting/locating/seeing/reading items or with minor screen size problems
4. Mostly clear, able to select/locate/see/ read items
5. Professional, simple, clear, orderly, logically organised, device display optimized. Every design component has a purpose

6. **General Graphics:** How high is the quality/resolution of graphics used for buttons/icons/menus/content of My Care Hub?

1. Graphic appear amateur, very poor visual design-disproportionate, completely stylistically inconsistent
2. Low quality resolution graphics; low quality visual design-disproportionate, stylistically inconsistent
3. Moderate quality graphics and visual design (generally consistent in style)
4. High quality/resolution graphics and visual design-mostly proportionate, stylistically consistent
5. Very high quality/resolution graphics and visual design-proportionate, stylistically consistent throughout

7. **Visual appeal:** How good does My Care Hub look?

1. No visual appeal, unpleasant to look at, poorly designed

2. Little visual appeal-poorly designed, bad use of colour.

3. Some visual appeal-average, neither pleasant nor unpleasant

4. High level of visual appeal-seamless graphics-consistent and professionally designed

5. As above + attractive, memorable, stands out; use of colour enhances app features/menu

8. **Graphic visual appeal of the analytics:** is the visual display of the log of graphs clear and understandable? Note, this refer to the analytic function called ‘‘view insight’’present in My Care Hub?

1. Graphic appear amateur, very poor visual design-disproportionate, completely stylistically inconsistent

2. Low quality/resolution graphics; low quality visual design-disproportionate, stylistically inconsistent

3. Moderate quality graphics and visual design (generally consistent in style)

4. High quality/resolution graphics and visual design-mostly proportionate, stylistically consistent

5. Very high quality/resolution graphics and visual design-proportionate, stylistically consistent throughout

9. **What is your overall rating of My Care Hub?**

1. * One of the worst apps have used

2. **

3. *** Average

4. ****

5. ***** One of the best apps have used

Additional Questions for participants who have diabetes

Satisfaction with My Care Hub for Diabetes Self-Management

1. **Usefulness**: Features of My care Hub are useful for diabetes self-management?

    1. Strongly Disagree
    2. Disagree
    3. Neither agree nor disagree
    4. Agree
    5. Strongly Agree

2. **Target group:** Is My Care Hub features appropriate for people with type 1 or type 2 diabetes?

    1. Completely inappropriate/unclear/ confusing
    2. Mostly inappropriate/unclear/confusing
    3. Acceptable but not targeted. May be inappropriate/unclear/confusing
    4. Well targeted, with negligible issues
    5. Perfectly targeted, no issues found

3. Quality of information: Is My Care Hub educational content well written, understandable and appropriate for people with type 1 or type 2 diabetes? This refers to the feature called "Educational tips" in My Care Hub and the messages under it.

    1. Irrelevant/inappropriate/incoherent/incorrect
    2. Poor. Barely relevant/appropriate/may be incorrect
    3. Moderately relevant/appropriate/coherent/correct
    4. Relevant/appropriate/coherent/correct
    5. Highly relevant, appropriate, coherent and correct

4. **App feedback**: Is the feedback messages in My Care Hub understandable and appropriate for your type of diabetes? This refers to the feedback (pop-up) messages displayed when blood glucose data is inputted into the app.

1. Irrelevant/inappropriate/incoherent/incorrect
2. Poor. Barely relevant/appropriate/may be incorrect
3. Moderately relevant/appropriate/coherent/correct
4. Relevant/appropriate/coherent/correct
5. Highly relevant, appropriate, coherent and correct

5. **Awareness:** My Care Hub educational information is useful for improving knowledge/awareness about the importance of diabetes self-management

1. Strongly Disagree
2. Disagree
3. Neither agree nor disagree
4. Agree
5. Strongly Agree

6. **Motivation:** The use of My Care Hub is likely to increase motivation of people with diabetes to engage in their self-management activities.

1. Strongly Disagree
2. Disagree
3. Neither agree nor disagree
4. Agree
5. Strongly Agree

7. **Intention to use:** If I have continual access to this app. I predict that I could use it.

1. Strongly Disagree

2. Disagree
3. Neither agree nor disagree
4. Agree
5. Strongly Agree

8. **Recommendation:** Would you recommend this app to people with type 1 diabetes (T1DM) or Type 2 Diabetes (T2DM)

1. I would not recommend this app to anyone with T1DM or T2DM
2. I could recommend this app to few people with T1DM or T2DM
3. I could recommend My Care Hub to several people with T1DM or T2DM
4. I could recommend My Care Hub to many people with T1DM or T2DM
5. I could recommend My Care Hub to anyone with T1DM or T2DM

General Impressions

1. Is there anything about My Care Hub that you like in particular?
   ……………………………………………………………………………………
   ……………………………………………………………………………………
2. Anything that you did not like. Anything that annoyed you?
   ……………………………………………………………………………………
   ……………………………………………………………………………………
3. Are there other observations you noted during your use of My Care Hub or you have some suggestions, please state it.
   ……………………………………………………………………………………
   ……………………………………………………………………………………

Thank you for taking the time to fill out this questionnaire
